# A general approach to visualize protein binding and DNA conformation without protein labelling

**DOI:** 10.1038/ncomms10976

**Published:** 2016-03-08

**Authors:** Dan Song, Thomas G. W. Graham, Joseph J. Loparo

**Affiliations:** 1 Harvard Biophysics Program, Harvard Medical School, Boston, Massachusetts 02115, USA; 2 Department of Biological Chemistry and Molecular Pharmacology, Harvard Medical School, 250 Longwood Avenue, Seeley G. Mudd Room 204B, Boston, Massachusetts 02115, USA; 3 Department of Systems Biology, Harvard Medical School, Boston, Massachusetts 02115, USA

## Abstract

Single-molecule manipulation methods, such as magnetic tweezers and flow stretching, generally use the measurement of changes in DNA extension as a proxy for examining interactions between a DNA-binding protein and its substrate. These approaches are unable to directly measure protein–DNA association without fluorescently labelling the protein, which can be challenging. Here we address this limitation by developing a new approach that visualizes unlabelled protein binding on DNA with changes in DNA conformation in a relatively high-throughput manner. Protein binding to DNA molecules sparsely labelled with Cy3 results in an increase in fluorescence intensity due to protein-induced fluorescence enhancement (PIFE), whereas DNA length is monitored under flow of buffer through a microfluidic flow cell. Given that our assay uses unlabelled protein, it is not limited to the low protein concentrations normally required for single-molecule fluorescence imaging and should be broadly applicable to studying protein–DNA interactions.

## Abstract (web summary)

Single-molecule imaging of protein-DNA association requires fluorescently labelled protein, which limits the protein concentration that can be used. Here the authors exploit protein induced fluorescent enhancement of DNA sparsely labelled with Cy3 to visualize protein binding and correlate it with changes in DNA conformation.

## Graphical abstract

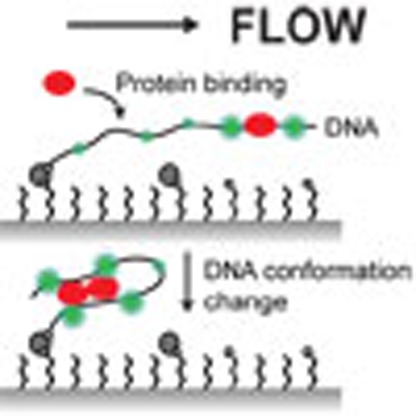

The interactions of proteins with DNA are central to the transmission and maintenance of genetic information. Many of these proteins alter the mechanical properties of DNA on binding. Proper chromosome condensation and segregation require DNA-binding proteins that facilitate chromosome organization through DNA bridging and bending^1^,^2^,^3^. In contrast, filament forming DNA-binding proteins involved in DNA replication and repair, such as the Rad51 recombinase, locally rigidify DNA^4^.

Ensemble approaches including surface plasmon resonance, white light interferometry and electrophoretic mobility shift assay are capable of measuring protein–DNA binding but are not sensitive to how these proteins change DNA conformation. For this reason, single-molecule nanomanipulation methods have been widely used to study how DNA-binding proteins remodel DNA^5^. In these approaches, DNA is weakly aligned by pulling on a bead attached to a DNA end, as in magnetic and optical tweezers^6^, or by exerting a hydrodynamic drag force on the DNA as done in flow stretching experiments^7^. Tweezer-based assays have higher spatial resolution and in the case of optical tweezers can physically move substrates within a microfluidic flow cell^8^. Flow-stretching methods such as DNA curtains^9^ allow for the observation of hundreds of substrates at a time. However, these nanomanipulation approaches only measure the change in extension of DNA as a proxy to investigate protein–DNA interactions. To directly visualize protein association on individual DNA molecules, one has to fluorescently label DNA-binding proteins and use fluorescence microscopy in combination with single-molecule nanomanipulation^10^,^11^,^12^,^13^. Such approaches can be challenging, owing to the increasing complexity of the instrumentation and difficulties in labelling and imaging labelled proteins. Labelling can inhibit protein activity and multiple labelling strategies may be attempted before an active construct is found. Moreover, imaging is usually limited to low concentrations of the labelled protein to allow for discrimination of the bound protein signal from the background fluorescence of freely diffusing proteins. Ultimately, this limits studies to proteins that have relatively strong binding affinities to DNA (*K*_d_<100 nM) (ref. 14).

Here we describe a single-molecule approach that simultaneously monitors protein association with DNA and changes in DNA conformation. Protein–DNA association is detected in the absence of protein labelling by exploiting protein-induced fluorescence enhancement (PIFE) of fluorescently labelled DNA that occurs on protein binding. For certain cyanine dyes, such as Cy3, which can undergo *cis*–*trans* isomerization on photoexcitation, increased local viscosity around the fluorophore due to protein binding results in increased quantum yield, brightness and fluorescence lifetime of the dye^15^. Fluorescent dyes that do not undergo this photoisomerization reaction, such as Cy3B^16^ and tetramethylrhodamine (TMR), are not expected to display PIFE. PIFE has distance sensitivity within a 0- to 4-nm range^16^ and it has been applied to study binding of individual proteins such as RIG-I and BamHI to short RNA or DNA oligonucleotides^16^,^17^. Our assay uses a 20-kb double-stranded DNA (dsDNA) substrate that is sparsely labelled with Cy3 dyes, allowing us to observe how DNA-binding proteins change the conformation of DNA, while simultaneously monitoring protein association.

We demonstrate the utility of visualizing protein binding and DNA conformation by characterizing how the *Bacillus subtilis* nucleoid-associated proteins Spo0J (ParB) and HBsu interact with DNA. Spo0J and HBsu perform critical roles in chromosome organization and segregation by changing DNA conformation^1^. We show that our assay can be used to measure the asynchronous binding and DNA compaction of Spo0J. Measurements probing the interaction of HBsu and DNA indicate that HBsu proteins continuously associate with DNA as they transition from a DNA bending to a DNA extension mode^18^,^19^,^20^. In addition, we show that DNA compaction by HBsu is sensitive to the force applied to DNA. Our approach is a relatively simple yet a highly quantitative method for characterizing complex protein–DNA interactions on kilobase length scales.

## Results

### Rationale and proof of principle

**Figure 1:**
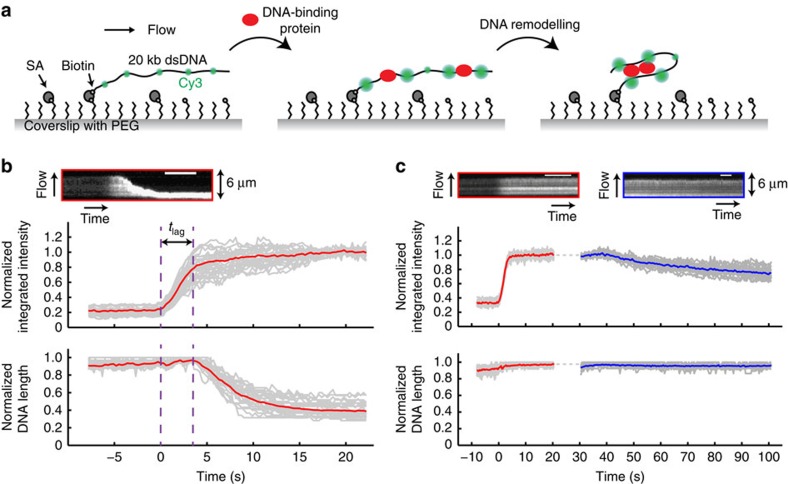
Rationale and validation of the single-molecule PIFE assay. (**a**) Left: a 20-kilobase (kb) dsDNA sparsely labelled with Cy3 dyes (green) is tethered to the surface of a functionalized glass coverslip and extended under a buffer flow. PEG, polyethylene glycol; SA, streptavidin. Middle: on addition of unlabelled DNA-binding proteins (red), fluorescence of the Cy3 dyes increases due to nearby protein binding and the resulting PIFE effect. Right: protein-induced changes in DNA conformation can be simultaneously monitored. Figures are not drawn to scale. (**b**) Binding of wild-type Spo0J (100 nM) to Cy3-labelled DNAs. Top: kymograph of a single DNA. Scale bar, 6 s. Bottom: trajectories of individual DNAs (grey) and the average over all trajectories (red). Integrated intensity and DNA length were normalized to the maximum values in individual trajectories. Time zero was defined as the starting point of protein association. *t*_lag_, lag time between protein binding and initiation of DNA compaction. (**c**) Interaction between Spo0J R82A (100 nM) and Cy3-labelled DNAs. Top: kymographs of a single DNA molecule showing protein association (red) under the same conditions as in **b** and dissociation after correction for photobleaching (blue) in binding buffer without any protein. Scale bar, 6 s. Bottom: trajectories of individual DNAs (grey) and average (red or blue). Dotted grey line indicates time when changing to the dissociation buffer after a brief stop in flow.

In our assay, 20 kb dsDNAs that were sparsely labelled with Cy3 dyes (one dye per kilobase on average; Supplementary Fig. 1) were immobilized at one end to a functionalized glass coverslip in a microfluidic flow cell (Fig. 1a). Buffer flow was applied to extend the DNAs and changes in the intensity and extension of the DNAs were imaged with total internal reflection fluorescence microscopy. On flowing in a buffer containing DNA-binding proteins, the integrated intensity of a Cy3-labelled DNA molecule is expected to increase due to protein binding and the resulting PIFE effect. Changes in the conformation of the DNA induced by associated proteins can be monitored at the same time by measuring the length of the fluorescently labelled DNA. Typically, 20–30 DNA molecules were imaged in a single field of view. These measurements can be readily multiplexed on a multi-channel flow cell, allowing for a number of different sample conditions to be tested in a relatively high-throughput manner. Importantly, we showed that the integrated intensities of Cy3-labelled DNAs did not vary over a wide range of salt concentrations (20–500 mM; Supplementary Fig. 2), indicating that changes in fluorescence intensity are strictly due to protein binding.

To validate our approach, we first looked at the ParB protein from *B. subtilis* known as Spo0J. ParB proteins are broadly conserved in bacteria and play important roles in both plasmid and chromosome segregation^2^,^21^,^22^. We previously demonstrated that wild-type Spo0J can bridge DNA and identified mutants, including Spo0J R82A, that are defective in DNA bridging but can still bind DNA with wild-type affinity^18^. A few seconds after introducing 100 nM wild-type Spo0J, the integrated intensity of each DNA molecule increased up to fivefold, indicating protein binding along the DNA (Fig. 1b and Supplementary Movie 1). Consistent with our prior observations using fluorescently labelled Spo0J^18^, DNA substrates were compacted all the way to the tether point, with a lag time between the initiation of protein association and the onset of compaction (Fig. 1b). In comparison, when flowing wild-type Spo0J over 20 kb TMR-labelled DNAs under identical buffer conditions, the integrated intensities of the DNAs increased by <1.5-fold, whereas the rate and extent of DNA compaction were identical to the Cy3-labelled DNAs (Supplementary Fig. 3). This demonstrates that the observed increase in intensity is mainly due to protein binding resulting from PIFE instead of DNA compaction.

Both the lag time and the rate of DNA compaction by wild-type Spo0J (Supplementary Fig. 4a,d) follow a Gaussian distribution (Supplementary Fig. 4b,e), and the average values were measured to be 3.3±0.1 s and 0.51±0.02 μm s^−1^ (mean±s.e.m.), respectively. These measurements are in good agreement with control experiments in which the extension of each DNA was tracked with a quantum dot (QD) tethered to the free end of DNA (see Methods and Supplementary Fig. 4c,f), demonstrating that the sparse Cy3 labelling of the DNA does not inhibit or alter protein binding.

In contrast to wild-type Spo0J, on addition of 100 nM Spo0J R82A in the same binding buffer, the integrated intensity of each Cy3-labelled DNA molecule increased by an average of threefold until reaching steady state, yet the DNAs remained fully extended, reflecting the inability of the mutant to compact DNA (Fig. 1c and Supplementary Movie 2). The initial rate of association to DNA for Spo0J R82A was indistinguishable from that of wild-type Spo0J (Supplementary Fig. 5). Although the fold increase in integrated intensity for the mutant and wild-type proteins were similar at the initiation of compaction (Supplementary Fig. 5), the maximal value for wild-type Spo0J is ∼1.5 times higher than that for Spo0J R82A (Supplementary Fig. 5), consistent with the stabilization of the wild-type protein on DNA due to the formation of higher-order bridging interactions.

To investigate protein dissociation from the DNA substrates, we then washed off Spo0J R82A with binding buffer without any protein (Fig. 1c). The integrated intensities of DNA molecules decreased over time at a rate that is much faster than photobleaching (Supplementary Fig. 6), indicating dissociation of the bound proteins, while the length of the DNA remained the same as that before washing (Fig. 1c). Increasing the concentration of salt in the washing buffer led to a faster decrease in fluorescence intensity, demonstrating that dissociation of Spo0J R82A from DNA is salt dependent (Supplementary Fig. 7).

### Measuring DNA-binding affinity at the single-DNA level

**Figure 2:**
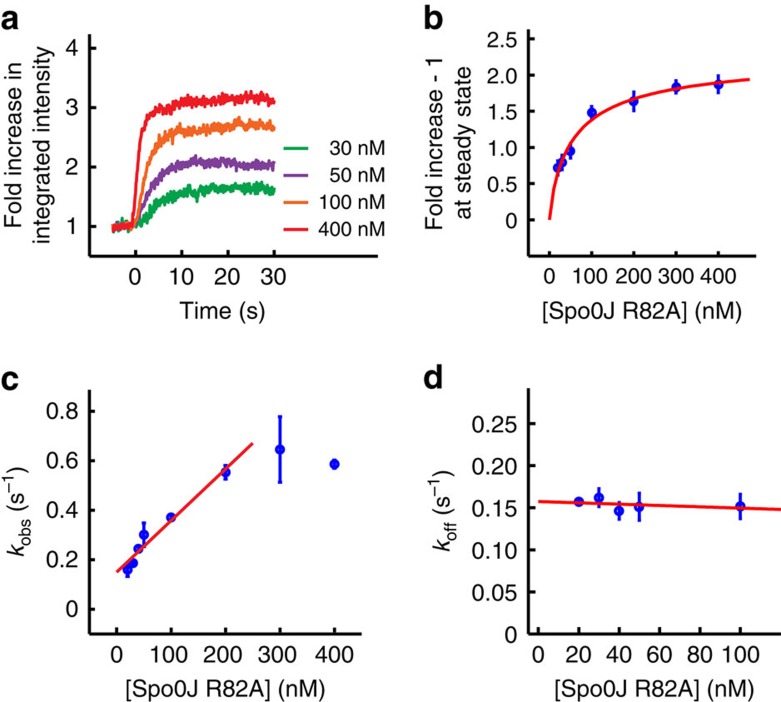
Measuring the binding affinity (*K*_d_) of the Spo0J R82A mutant on individual DNA molecules. (**a**) Trajectories of integrated intensity measuring association of Spo0J R82A at indicated concentrations to Cy3-labelled DNAs in binding buffer containing 150 mM NaCl. Each trajectory was averaged over 20–30 DNAs. Fold increase in integrated intensity was calculated by dividing each trajectory by the value averaged for the first few seconds before protein binding. Time zero was defined as the starting point of protein association. (**b**) Fold increase in integrated intensity at steady state after subtracting the baseline (onefold) fitted with a Hill equation (see Methods). (**c**) Observed rate constant for association (*k*_obs_) and linear fit (red line), to obtain the rate constant for association (*k*_on_). (**d**) Rate constant for dissociation (*k*_off_) and linear fit (red line). The average value was estimated from the *y* intercept, as the slope does not significantly differ from zero. All data points shown in **b**–**d** are mean±s.e.m. between at least three replicates.

By observing protein association, we can quantitatively characterize protein–DNA interactions using both steady-state measurements and real-time kinetics, which we demonstrated for Spo0J R82A. First, we observed a greater fold increase in integrated intensity at steady state with increasing protein concentration (Fig. 2a). Similar effects were also seen for the wild-type protein at low concentrations (Supplementary Fig. 8). The Spo0J R82A steady-state fold increase in integrated intensity could be well described by a Hill equation (see Methods) with an apparent *K*_d_*=*60.5±27.6 nM (fit±error estimate) and a Hill coefficient *n=*0.8±0.2 (Fig. 2b). Alternatively, real-time kinetic parameters can be extracted by fitting the association and dissociation intensity trajectories with the Langmuir binding model (see Methods and Supplementary Fig. 9), assuming Spo0J R82A interacts with each DNA-binding site in a 1:1 ratio. At lower protein concentrations (≤200 nM), the observed rate constant for association (*k*_obs_) is linearly proportional to protein concentration: *k*_obs_*=k*_on_ [protein]+*k*_off_, where *k*_on_ and *k*_off_ are rate constants for association and dissociation, respectively. Linear fitting *k*_obs_ versus protein concentration (Fig. 2c) yielded a slope (that is, *k*_on_) of 2.1±0.2 × 10^6^ M^−1^ s^−1^. At higher protein concentrations (≥300 nM) *k*_obs_ saturates, because protein binding to DNA occurs at a rate that is too fast to be resolved with our assay (Fig. 2c). By fitting the dissociation trajectories with an exponential decay function (Supplementary Fig. 9b), *k*_off_ was measured to be independent of protein concentration up to 100 nM (Fig. 2d), with an average value of 0.16±0.01 s^−1^. Higher concentrations of Spo0J R82A (≥300 nM) weakly compacted DNA (Supplementary Fig. 10), while subsequent washing resulted in a substantial decrease in the rate of protein dissociation. By taking the ratio between *k*_off_ and *k*_on_, we estimated the *K*_d_ to be 76.2±8.7 nM, which is in good agreement with the steady-state measurements.

As a point of comparison to ensemble methods that measure protein affinities, we used white light interferometry to obtain the apparent DNA-binding affinity of Spo0J R82A to a 39-bp dsDNA with a scrambled *parS*-binding site^18^ under the same binding conditions (see Methods and Supplementary Fig. 11). The equilibrium binding responses for different concentrations of Spo0J R82A (Supplementary Fig. 11a) can be described by the Hill equation with an apparent *K*_d_*=*130.8±24.3 nM and a Hill coefficient *n=*1.9±0.5 (Supplementary Fig. 11b). The nonspecific DNA-binding affinities of Spo0J R82A are similar between our single-molecule and bulk measurements, whereas the greater cooperativity we observe in the white light interferometry experiments is probably due to proposed nearest-neighbour interactions^23^,^24^ that occur more readily on short DNA substrates.

### Visualization of HBsu switching between DNA-binding modes

HU (histone-like protein from *Escherichia coli* strain U93) is a small and abundant chromosomal architectural protein^25^ that plays important roles in gene regulation, DNA replication, recombination and repair^26^,^27^. Previous studies on HU using magnetic tweezers^19^,^20^ and atomic force microscopy imaging^20^,^28^ showed that HU bends DNA at low protein concentrations but forms a nucleoprotein filament at higher protein concentrations (≥600 nM) that extends DNA. This bimodal binding behaviour is sensitive to salt concentration^29^ and has been observed in single-molecule studies with the *B. subtilis* homologue HBsu^18^. Our assay, which can simultaneously monitor protein association and changes in the DNA conformation, can directly probe the transition between these HBsu–DNA binding modes.

**Figure 3:**
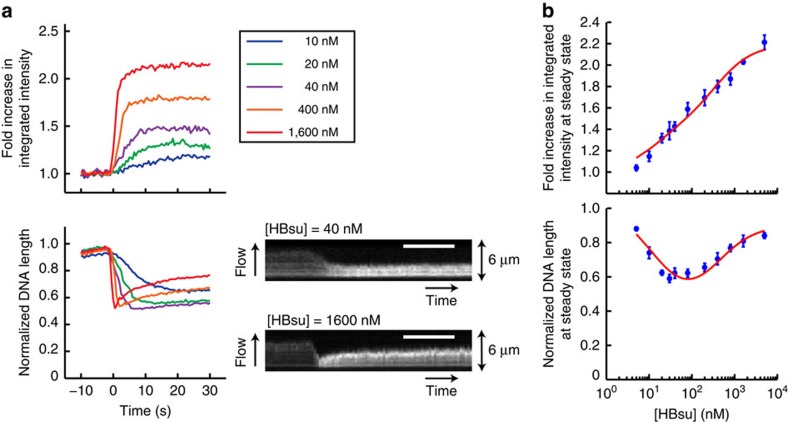
Dual binding modes of HBsu revealed by the single-molecule PIFE assay. (**a**) Trajectories and kymographs showing interactions between HBsu at indicated concentrations and the Cy3-labelled DNAs. Each trajectory was averaged over 20–30 DNAs. Fold increase in integrated intensity (top) and normalized DNA length (bottom) were calculated by dividing each trajectory by the values averaged for the first few seconds before protein binding. Time zero was defined as the starting point of protein association. Scale bar, 10 s. (**b**) Steady-state measurements of fold increase in integrated intensity (top) and in DNA length (bottom) at different concentrations (log-scale) of HBsu, fitted with a two-state binding model (red line). All data points shown are mean±s.e.m. between at least three replicates.

Given that our assay uses unlabelled proteins, we were able to observe HBsu association with DNA at high protein concentrations (≥1 μM) without concern for increasing the background intensity that is often limiting in fluorescence microscopy. We found that after introducing HBsu into the flow cell, DNA was compacted efficiently, coincident with protein association (Fig. 3a and Supplementary Fig. 4c,f), and then re-extended at higher protein concentrations (≥200 nM) (Fig. 3a and Supplementary Movie 3). Interestingly, the fold increase in integrated intensity at steady state continued to increase with protein concentration even over the concentration regime in which DNA length was largely unchanging (∼20 to 100 nM) (Fig. 3b). This suggested a simple binding model (see Methods and Supplementary Fig. 12) in which initial binding of HBsu to high-affinity sites (*K*_1_≈13 nM) compacts DNA by local bending, while subsequent binding to low-affinity sites (*K*_2_≈390 nM) re-extends DNA by formation of a filament. This model provided a good fit to the steady-state increase in fluorescence intensity and changes in DNA extension (see red lines in Fig. 3b). Such a model is consistent with results from fluorescence resonance energy transfer experiments with *E. coli* HU, which demonstrated multiple DNA-binding modes with various footprints^30^.

As our assay is based on flow-stretching DNAs, tension varies along the length of the DNA, which may affect protein binding differentially along the molecule. Using site-specific labelling of DNA (see Methods and Supplementary Fig. 13), an approach we termed DNA motion capture^18^, we measured the differential extension of segments along a 24-kb DNA and estimated the tension^31^. At a flow rate of 100 μl min^−1^, the tension was ≥1 pN at the tether point and ≤0.1 pN close to the free end (Supplementary Fig. 13c). Interestingly, we observed that at steady state the fluorescence intensities along the length of a Cy3-labelled DNA compacted by HBsu monotonically increased from the tether point to the free end of the DNA (Supplementary Fig. 14b). In contrast, the fluorescence intensities along the length of a Cy3-labelled DNA associated with Spo0J R82A, which could not compact DNA at steady state, was uniformly distributed, although at higher values than that of a naked DNA due to PIFE (Supplementary Fig. 14c). Our observations thus suggested that compaction driven by HBsu was not uniform along a flow-stretched DNA molecule: DNA was more readily compacted at the free end where the tension was smaller and the fractional occupancy of HBsu might be higher (Supplementary Fig. 14b). This is in agreement with previous magnetic tweezer experiments, which argued for a force-dependent DNA binding of *E. coli* HU^32^.

**Figure 4:**
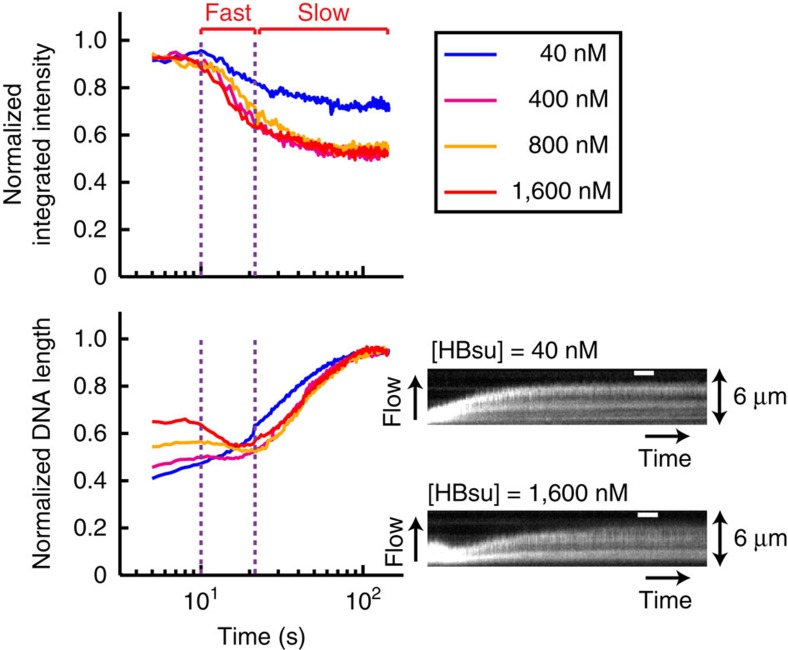
Biphasic dissociation of HBsu. Trajectories (log-scale in time) and kymographs showing dissociation of HBsu at indicated concentrations from the Cy3-labelled DNAs when washing with binding buffer without any protein. Each trajectory was corrected for photobleaching and averaged over 20–30 DNAs. Integrated intensity (top) and DNA length (bottom) were normalized by the maximum values. Time zero was defined as the starting point of protein dissociation and was shifted by 10 s for visualization. Scale bar, 10 s. Purple dotted lines indicate transitions from the fast to the slow dissociation phase for high protein concentrations.

We also investigated dissociation of HBsu from DNA by washing protein–DNA complexes with binding buffer without any protein (Fig. 4). At high protein concentrations (≥400 nM), dissociation occurred in two phases (Fig. 4 and Supplementary Movie 4): extended DNAs first re-compacted, coincident with a rapid decrease in protein association as measured by PIFE. DNAs then slowly re-extended to the contour length of naked DNA, coincident with slower protein dissociation (Fig. 4). In contrast, HBsu at lower protein concentrations only displayed monophasic dissociation and decompaction trajectories (see blue line in Fig. 4). The biphasic transition in the conformation of DNA that was coated with HBsu was also sensitive to force (Supplementary Fig. 15), in agreement with previous observations^29^. More importantly, the difference in the kinetics between the two dissociation phases further suggests that the extension mode of HBsu is less stable than the bending mode, as the nucleoprotein filament collapsed more rapidly during washing.

## Discussion

Our method to simultaneously detect protein association and DNA conformation provides advantages over other experimental approaches that measure only binding kinetics (surface plasmon resonance, white light interferometry and electrophoretic mobility shift assay) or DNA conformation (optical or magnetic tweezers). Here we have applied our approach to two important bacterial DNA-binding proteins involved in chromosome condensation and segregation (Spo0J and HBsu), yet our assay could, in principle, be used to study any DNA-binding protein. We have demonstrated that PIFE resulting from protein binding to DNA is an effective proxy for measuring protein association and dissociation, and that by quantitatively correlating protein binding with changes in DNA extension, our approach can provide detailed molecular insight into how proteins interact with DNA.

Importantly, our method obviates the often difficult and time-consuming task of fluorescently labelling a protein without affecting its function. Imaging of protein–DNA interactions with our assay is not limited to low protein concentrations, as is often the case for fluorescently labelled proteins due to high background fluorescence resulting from freely diffusing labelled proteins. By using buffer flow to extend DNAs, we are able to probe force-dependent DNA compaction with a simple setup in a relatively high throughput manner. Extension of these measurements to a multi-channel microfluidic device will enable rapid screening of experimental conditions. As a proof of principle, we showed how mutants deficient in DNA compaction but not DNA binding (for example, Spo0J R82A) could be identified and how our approach could facilitate screening for such mutants.

## Methods

### DNA construction

*Single-molecule PIFE experiments*. A 20-kb segment of genomic DNA from *B. subtilis* strain PY79 was first PCR amplified using primers oTG489 and oTG491 (Supplementary Table 1). Primers oTG437 and oTG488 (Supplementary Table 1), which are complementary to adapter sequences in oTG489 and oTG491, were then used in a second round of PCR amplification to generate DNAs with either biotin or digoxigenin end modifications. Fluorescent labels were randomly incorporated by including Cy3-dUTP (Enzo Life Sciences) for the PIFE experiments or TMR-6-dUTP (Perkin Elmer) for the control experiments in the second PCR mix at 1/30 the molar concentration of the unlabelled nucleotides. The 20-kb PCR product was then gel purified using the QIAEX II Gel Extraction Kit (Qiagen). All PCR was done in a 25-μl reaction using the LongAmp *Taq* DNA polymerase (NEB).

*DNA motion capture assay*. Biotinylated bacteriophage λ genomic DNA (NEB) was first constructed by annealing and ligating phosphorylated oligonucleotide BL2 (Supplementary Table 1) to the single-stranded *cos* site at one end of the *λ* DNA. Biotin-*λ* DNA was then digested with XbaI (NEB) at 37 °C for at least 3 h, to generate a biotinylated 24-kb DNA containing four EcoRI binding sites (3,530, 9,334, 16,755 and 22,398 bp from the tether point). Labelling of the biotinylated 24 kb DNA with 605-nm QDs (Life Technologies) conjugated to His6-EcoRI^E111Q^ protein^18^ was done by preincubating the DNA with the anti-His6 QDs in EcoRI binding buffer (20 mM Tris at pH 7.5, 100 mM NaCl, 100 μg ml^−1^ BSA) for at least 30 min at room temperature^18^.

### Protein expression and purification

Wild-type and mutant Spo0J proteins and HBsu were expressed with an amino-terminal His6-SUMO tag in BL21 cells and purified via Ni-NTA column (Qiagen) using a two-step affinity method as previously described^18^. Briefly, peak fractions of His6-SUMO fusion proteins were collected after running through the Ni-NTA column and were dialysed overnight with His6-Ulp1 protease^18^, to cleave off the His6-SUMO tag. The cleaved tag was then removed from the proteins with Ni-NTA resin on the second day. Spo0J proteins were further purified using ion-exchange chromatography with SP-sepharose^18^.

### Flow cell and single-molecule imaging

Flow cells were assembled as previously described^33^. Briefly, five 2 × 10 mm rectangular channels were cut out of double-sided tape (Grace Bio-Labs, 0.12 mm thick), which was sandwiched between a quartz microscope slide (Technical Glass) and a functionalized glass coverslip. The coverslip surface was passivated with polyethylene glycol and coated with streptavidin to tether biotinylated DNA substrates. The quartz top was drilled with two holes above each channel, to accommodate 4- or 7-cm long inlet and 3-cm long outlet polyethylene tubing (Intramedic, PE60). The shorter inlet was used to minimize mixing time of solutions when measuring the rate constants for protein association (*k*_obs_) and dissociation (*k*_off_). A laminar flow provided by an automated syringe pump (Harvard Apparatus) was applied to extend the DNA and deliver solutions into the flow cell.

Cy3- or TMR-labelled DNAs and QD-labelled DNAs were imaged on a homebuilt through-objective total internal reflection fluorescence microscope^18^. Fluorescence was excited using a 532-nm laser (Coherent, Compass 215M-75) through a high-numerical aperture objective (Olympus, PlanApo × 60, numerical aperture 1.45) on an inverted microscope (Olympus, IX71). Emitted fluorescence was collected through the same objective after passing through a dichroic mirror (Chroma, Z532RDC) and an emission filter (Chroma, HQ600/75 M) before × 1.6 magnification onto an electron multiplying charge-coupled device (Hamamatsu, EM-CCD 9100–13). Images were acquired at 10 Hz with continuous exposure. A low incident laser power (∼2 mW) was used to minimize photobleaching.

### Single-molecule PIFE experiments

The coverslip surface of the flow cell coated with streptavidin was first washed and incubated with the biotinylated and Cy3- or TMR-labelled 20-kb DNAs (10–15 pM) in a blocking buffer (20 mM Tris at pH 7.5, 100 mM NaCl and 100 μg ml^−1^ BSA) for ∼5 min or until a sufficient number of tethered DNAs was observed. Unbound DNAs were then washed away with binding buffer (20 mM Tris at pH 7.5, 100 μg ml^−1^ BSA and 100 mM NaCl unless indicated otherwise). All types of proteins were diluted to the indicated concentrations with the binding buffer before being continuously introduced into the flow cell at a flow rate of 100 μl min^−1^. To image protein dissociation, flow was briefly stopped (10–20 s) to exchange to the washing buffer, which was identical to the binding buffer, unless indicated otherwise, but without protein. A different field of view was used to image protein dissociation after imaging protein association, to minimize photobleaching.

### PIFE data analysis

Trajectories of integrated fluorescence intensity and length of the Cy3- or TMR-labelled DNAs were analysed using custom scripts written in MATLAB. Briefly, 20–30 regions of interest (ROIs) containing single DNA molecules were first marked manually in ImageJ. Integrated intensity of each ROI over time after subtracting background fluorescence was calculated automatically in MATLAB. To measure DNA length, one-dimensional projections of each ROI onto the axis along the flow direction were aligned to generate a kymograph. DNA length was then measured by counting the number of pixels in each frame above an intensity threshold determined using the kymograph.

### Measuring kinetics of DNA compaction using a QD end label

An unlabelled 20-kb dsDNA was constructed in the same way as the Cy3-labelled DNA, except unlabelled dUTP instead of the Cy3-dUTP was included in the second round of PCR amplification. DNA was tethered to the streptavidin-coated surface of the flow cell at the biotinylated end, while the free end of the DNA was labelled by flowing in carboxyl-coated 605-nm QDs (Life Technologies) that had been conjugated to anti-digoxigenin antibodies (Roche), following the manufacturer's instructions. A small amount of tracer dye was added in the binding buffer to monitor arrival of unlabelled protein. The position of each QD was determined from a Gaussian fit of the one-dimensional projection onto the axis parallel to the flow direction using custom scripts written in MATLAB.

### White light interferometry DNA-binding experiments

Oligonucleotides oTG044F and oTG043R (Supplementary Table 1) were annealed in 1 × TE+50 mM NaCl by heating at 95 °C for 2 min and slowly cooled to room temperature. Streptavidin biosensors (ForteBio) were incubated with binding buffer (20 mM Tris at pH 7.5, 150 mM NaCl, 100 μg ml^−1^ BSA and 0.02% Tween-20) for at least 10 min before the experiments. Binding kinetics were measured on a BLItz instrument (ForteBio) using the BLItz Pro software (ForteBio), following the manufacturer's instructions. Briefly, 4 μl of annealed biotinylated oligo duplex (1 μM) was loaded on a streptavidin biosensor for 2 min. A baseline of binding response was then determined by incubating the sensor with 250 μl of binding buffer without protein for 30 s. Two hundred and fifty microlitres of Spo0J R82A protein diluted to the indicated concentrations in the binding buffer was then used to measure protein association until reaching steady state (for a duration of 1,000 s). The same buffer from the baseline step was used to measure protein dissociation until the binding response decreased by at least 50% (for a duration of 300 s).

### Kinetic model for Spo0J R82A binding to DNA

The binding kinetics of Spo0J R82A were assumed to follow a simple 1:1 Langmuir model:


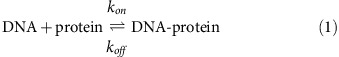


where protein association is governed by a forward rate constant *k*_on_ and protein dissociation is governed by a reverse rate constant *k*_off_. Given the vast excess of protein to DNA under our experimental conditions, it was assumed that the concentration of free protein in the reaction was effectively unchanged as the DNA–protein complex formed. Therefore, the rate constant *k*_on_ can be related to the observed rate constant for association *k*_obs_ using the following^34^:


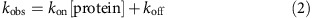


where [protein] indicates concentration of Spo0J R82A (monomer). Solving the associated differential equations for the association and dissociation phases gives:


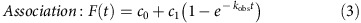



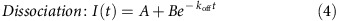


where *F*(*t*) is fold increase in the integrated intensity of the Cy3-labelled DNAs over time during the association phase. The fitting parameters *c*_0_ and *c*_1_ describe the minimum binding signal *F*_min_ at *t=*0 (*F*_min_*=c*_0_) and the maximum binding signal *F*_max_ at steady state (*F*_max_*=c*_0_+*c*_1_) (Supplementary Fig. 9a). *I*(*t*) is integrated intensity of the protein-bound Cy3-labelled DNAs normalized by the intensity at the beginning of the dissociation phase (Supplementary Fig. 9b). The fitting parameters *A* and *B* describe the maximum intensity *I*_max_ at *t=*0 (*I*_max_*=A*+*B*) and the minimum intensity *I*_min_ when the exponential decay function reaches a plateau at long times (*I*_min_*=A*). Before fitting the dissociation trajectories, photobleaching was corrected as the following:


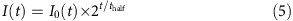


where *I*_0_(*t*) is integrated intensity of the Cy3-labelled DNA without normalization before correcting for photobleaching and *t*_half_ is the half-life of photobleaching (*t*_half_≥300 s; Supplementary Fig. 6).

### Determination of apparent *K*_d_ using steady-state measurements

The steady-state binding signal from the single-molecule PIFE assay was obtained by fitting the data with equation (3) (*F*_max_*=c*_0_+*c*_1_) (Supplementary Fig. 9). For the white light interferometry experiments, the steady-state binding response was determined by averaging over the saturating values for the last 200 s during the association phase (Supplementary Fig. 11a). The apparent *K*_d_ in both the single-molecule PIFE (Fig. 2b) and the white light interferometry experiments (Supplementary Fig. 11b) was determined by fitting the steady-state data to the Hill equation as the following:


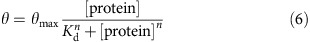


where *θ* is the measured binding signal, *θ*_max_ is the maximum binding signal, *n* is the Hill coefficient and [protein] indicates concentration of Spo0J R82A (monomer).

### Two-state binding model for HBsu

A DNA molecule was modelled as an infinite string of segments (Supplementary Fig. 12), each of which could bind zero, one or two HBsu dimers:


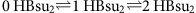


The model contained six free parameters: equilibrium constants for sequential binding of the first and second HBsu dimer (*K*_1_ and *K*_2_), and fold change of integrated intensity and DNA length of the two HBsu bound states, relative to the unbound state (*I*_1_, *I*_2_, *L*_1_ and *L*_2_). The fold change of intensity and length of the whole DNA are given by:


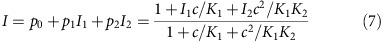



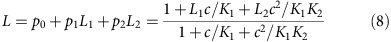


where *c* is the concentration of HBsu and *p*_0_, *p*_1_ and *p*_2_ are the proportions of segments bound to 0, 1 or 2 HBsu dimers (where *p*_0_+*p*_1_+*p*_2_=1). Using the MATLAB fmincon function, fold change in intensity and DNA length as a function of HBsu concentration were fit simultaneously to the above two equations, to extract the parameters *K*_1_, *K*_2_, *I*_1_, *I*_2_, *L*_1_ and *L*_2_.

### Estimating tension on flow-stretched DNAs

EcoRI^E111Q^-QD-labelled 24-kb DNAs were tethered to the streptavidin-coated surface of a flow cell at the biotinylated end and were extended under a buffer flow (20 mM Tris at pH 7.5, 100 mM NaCl and 100 μg ml^−1^ BSA) at indicated flow rates (Supplementary Fig. 13a). DNAs were poststained with SYTOX, to determine the position of the tether point. Subpixel position of each QD from the tether point was determined from a Gaussian fit of the one-dimensional projection onto the axis parallel to the flow direction using custom scripts written in MATLAB (Supplementary Fig. 13b). Differential extension (*ξ*) of a flow-stretched DNA between the QD sites (Supplementary Fig. 13c) was calculated based on the following definition^31^:


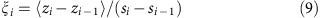


where 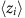 is the average distance of QD *i* from the tether point and *i* runs from 1 to 4, with *i=*1 closest to the tether point. *s*_*i*_ is the location of the *i*th QD site along the contour of the DNA and *s*_*i*_=*L* × *N*_*i*_/*N*_total_, where *N*_*i*_ is the sequence position of the *i*th EcoRI site as measured from the tether point, *N*_total_*=*23,994 for the XbaI digested *λ* DNA and *L* is the full contour length of the DNA. *z*_0_*=s*_0_*=*0 at the tether point. The tension on each DNA segment was estimated using the force-extension curve fitted with a worm-like chain model^31^.

### Code availability

MATLAB code files are available from the authors on request.

## Additional information

**How to cite this article:** Song, D. *et al.* A general approach to visualize protein binding and DNA conformation without protein labelling. *Nat. Commun.* 7:10976 doi: 10.1038/ncomms10976 (2016).

## Supplementary Material

Supplementary Information Supplementary Figures 1-15, Supplementary Table 1 and Supplementary Reference

Supplementary Movie 1 Compaction of 20 kilobase (kb) Cy3-labeled dsDNAs by wild-type Spo0J (100 nM), corresponding to Fig. 1b. Only a quarter of the field of view is shown. Movie is at 2× speed. Time zero was defined as the starting point of protein association (time stamps). DNA molecules highlighted with yellow boxes were analyzed for Fig. 1b. Sheared Cy3-labeled DNAs that were shorter than 20 kb appeared as spots on the surface. Scale bar is 5 μm.

Supplementary Movie 2 Interaction between Spo0J R82A (100 nM) and 20 kb Cy3-labeled dsDNAs, corresponding to Fig. 1c. Only a quarter of the field of view is shown. Movie is at 2× speed. Time zero was defined as the starting point of protein association (time stamps). DNA molecules highlighted with yellow boxes were analyzed for Fig. 1c. Sheared Cy3-labeled DNAs that were shorter than 20 kb appeared as spots on the surface. Scale bar is 5 μm.

Supplementary Movie 3 Single-molecule PIFE imaging reveals the dual binding modes of HBsu (1.6 μM), corresponding to Fig. 3. Only a quarter of the field of view is shown. Time zero was defined as the starting point of protein association (time stamps). DNA molecules highlighted with yellow boxes were analyzed for Fig. 3. Sheared Cy3-labeled DNAs that were shorter than 20 kb appeared as spots on the surface. Scale bar is 5 μm.

Supplementary Movie 4 Biphasic dissociation of HBsu (1.6 μM), corresponding to Fig. 4. The same field of view as shown in Supplementary Movie 3 was presented here. Movie is at 2x speed. Time zero was defined as the starting point of protein dissociation (time stamps). DNA molecules highlighted with yellow boxes were analyzed for Fig. 4. Sheared Cy3-labeled DNAs that were shorter than 20 kb appeared as spots on the surface. Scale bar is 5 μm.
