# Supplementary material for: A general approach to visualize protein binding and DNA conformation without protein labelling
Source: Nat Commun. 2016 Mar 8;7:10976. doi: 10.1038/ncomms10976 (PMC4786781; doi:10.1038/ncomms10976)
Supplement: Supplementary Information — Supplementary Figures 1-15, Supplementary Table 1 and Supplementary Reference [file ncomms10976-s1.pdf]

SUPPLEMENTARY FIGURES

Supplementary Figure 1

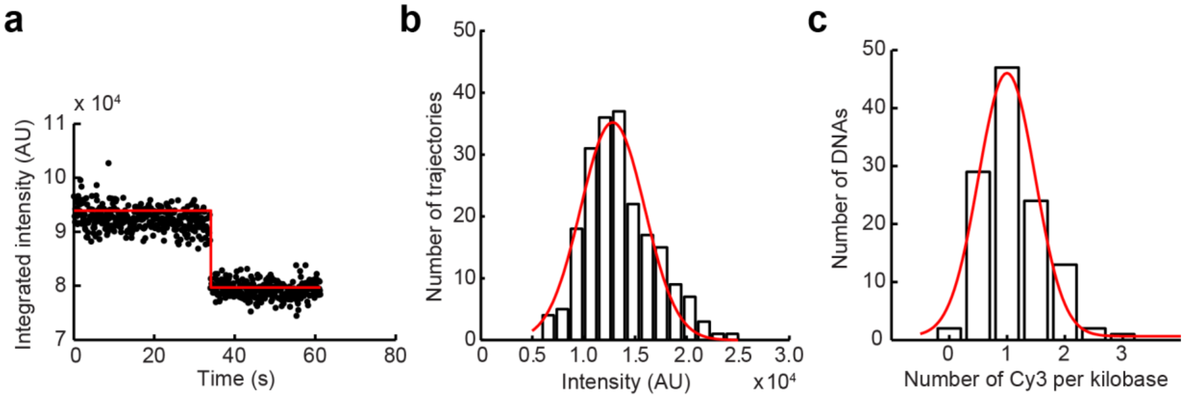

Supplementary Figure 1: Quantification of the average number of Cy3 dyes per labeled DNA. (**a**) A representative trajectory showing a photobleaching step of a biotinylated 100 bp DNA duplex labeled with a single Cy3 dye bound to the surface of a glass coverslip. The change in intensity of each photobleaching step was obtained by fitting with a step function. (**b**) Histogram of photobleaching step size fitted with a Gaussian distribution (red) (mean ± s.d.: 1.3 ± 0.3 × 10^4 AU). The mean value was used to calibrate the background subtracted integrated intensity of individual Cy3-labeled DNAs measured under the same imaging conditions. (**c**) Histogram of the calculated number of Cy3 dyes per DNA normalized by the DNA length (kilobase) and fitted with a Gaussian distribution (red) (mean ± s.d.: 1.00 ± 0.50 per kilobase).

Supplementary Figure 2

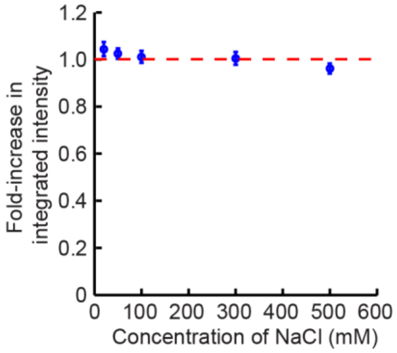

Supplementary Figure 2: The fold-increase in integrated intensity of a Cy3-labeled DNA molecule is independent of salt concentration. DNAs were washed with binding buffer at indicated concentrations of NaCl and imaged for about a minute. Fold-increase was calculated by taking the ratio of fluorescence intensities at the beginning and end of imaging. Data shown are mean ± s.e.m. calculated over 15 individual DNAs.

Supplementary Figure 3

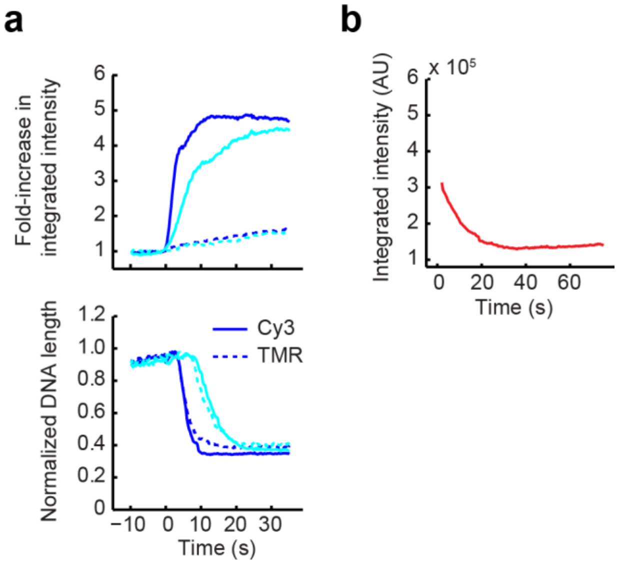

Supplementary Figure 3: Interactions between wild-type Spo0J and Cy3- or TMR-labeled DNAs. (**a**) Trajectories measuring association of 100 nM (blue) and 50 nM (cyan) wild-type Spo0J to either Cy3 (solid lines) or TMR (dotted lines) labeled DNAs. Each trajectory was averaged over 15 – 20 DNAs. Fold-increase in integrated intensity (top) and normalized DNA length (bottom) were calculated by dividing each trajectory by the values averaged for the first few seconds prior to protein binding. Time zero was defined as the starting point of protein association. (**b**) Photobleaching curve averaged over multiple TMR-labeled DNAs showing a multi-exponential decay, which is similar to previous observations[1]. Prior to imaging protein association, TMR-labeled DNAs were pre-bleached for ~30 seconds to minimize the fast photobleaching component.

Supplementary Figure 4

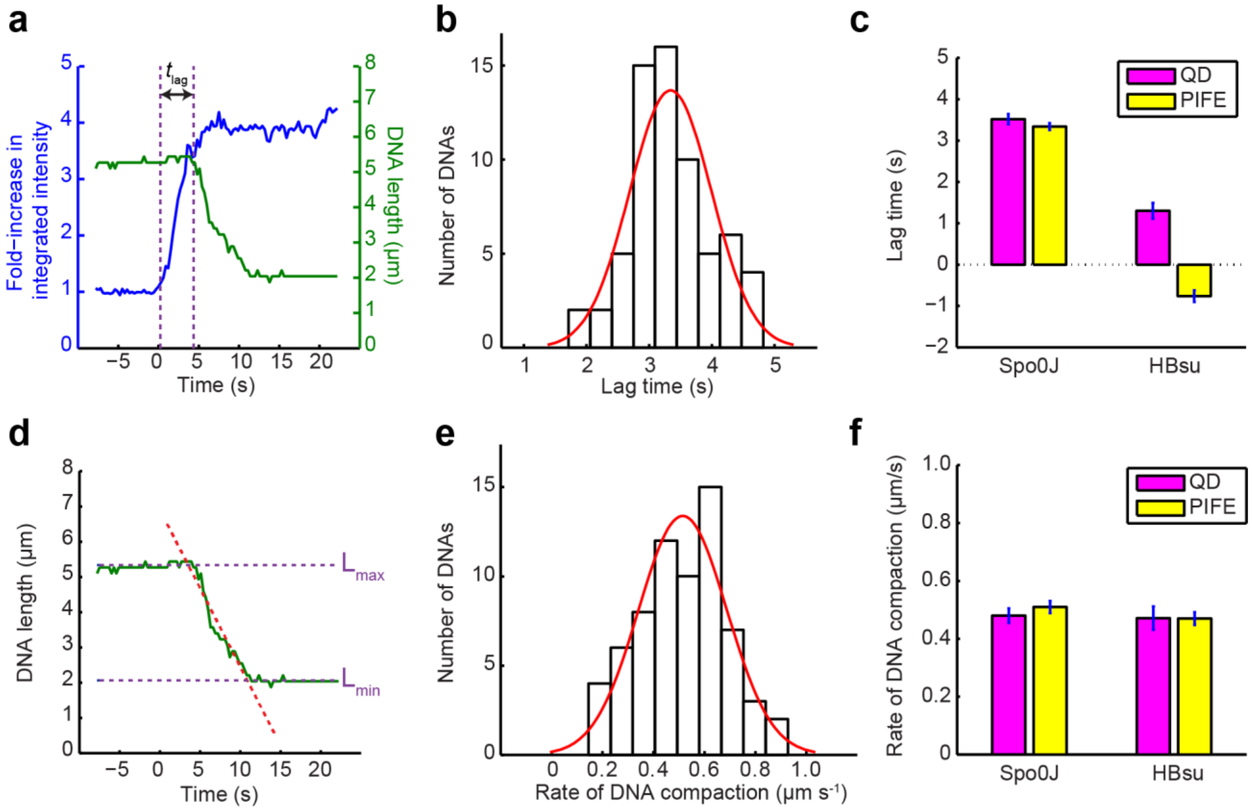

Supplementary Figure 4: Quantification of the kinetics of DNA compaction by either Spo0J (100 nM) or HBsu (40 nM). (**a**) Trajectories of fold-increase in integrated intensity (blue) and DNA length (green) of a single Cy3-labeled DNA molecule compacted by Spo0J. Time zero was defined as the starting point of protein association. Lag time (t_{lag}) is the time between protein binding and initiation of DNA compaction. t_{lag} = 4.17 s as shown here. (**b**) Histogram of lag times fitted with a Gaussian distribution (red) for Spo0J. (**c**) Comparison of the mean lag time measured in either the single-molecule PIFE assay (pink) or control experiments (yellow) in which extension of a DNA molecule was tracked with a quantum dot tethered to the free end of DNA (see Methods). For the control experiments protein arrival was tracked with a tracer dye in solution. Data shown are mean ± s.e.m. measured over ≥ 20 DNAs. It should be noted that the mean lag time of HBsu measured in the single-molecule PIFE assay was slightly less than zero, indicating that changes in DNA length were observable prior to protein association. This is likely due to the sparse labeling of DNA with Cy3 dyes and the short distance range of PIFE. (**d**) Trajectory of DNA length of a single Cy3-labeled DNA molecule compacted by Spo0J. Rate of DNA compaction was estimated by linear fitting the trajectory between maximum and minimum DNA lengths. Slope shown here is 0.45 ± 0.02 μm s^{-1} (fit ± error estimate). (**e**) Histogram of rates of DNA compaction fitted with a Gaussian distribution (red) for Spo0J. (**f**) Comparison of the mean rate of DNA compaction measured in either the single-molecule PIFE assay (pink) or the quantum dot control experiments (yellow). Data shown are mean ± s.e.m. measured over ≥ 20 DNAs.

Supplementary Figure 5

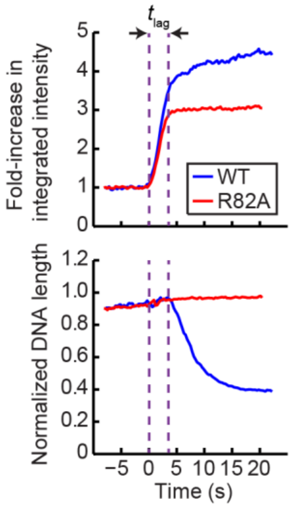

Supplementary Figure 5: A comparison of DNA binding kinetics between wild-type Spo0J (100 nM; blue) and Spo0J R82A (100 nM; red) under the same binding conditions. Trajectories shown here were averaged over 20 – 30 DNAs. Fold-increase in integrated intensity and normalized DNA length were calculated by dividing each trajectory by the values averaged for the first few seconds prior to protein binding. Time zero was defined as the starting point of protein association. t_{lag}: lag time between protein binding and initiation of DNA compaction.

Supplementary Figure 6

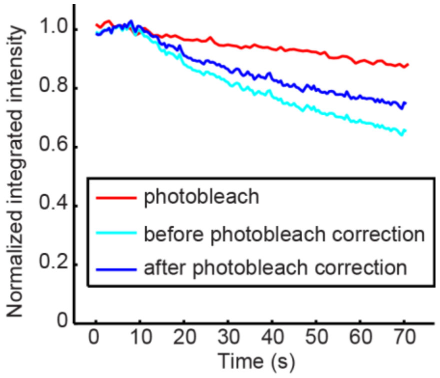

Supplementary Figure 6: Correction of dissociation kinetics for photobleaching. Photobleaching of the Cy3-labeled DNAs (red) was determined under matched imaging conditions but in the absence of protein. Each trajectory was averaged over 20 – 30 DNAs. Integrated intensity was normalized by the maximum value. The half-life of photobleaching was measured to be ≥ 300 s. Trajectory of protein dissociation after correcting for photobleaching (blue) is the same as shown in Fig. 1c.

Supplementary Figure 7

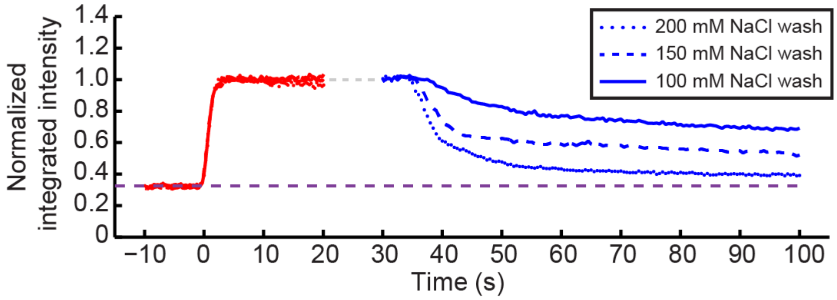

Supplementary Figure 7: Dissociation of Spo0J R82A from 20 kb Cy3-labeled DNAs is salt-dependent. In each experiment, a solution of Spo0J R82A (100 nM) in binding buffer with 100 mM NaCl was first flowed into the channel to image association with tethered DNAs (red trajectories). After a brief stop in flow (indicated by the dotted gray line), the DNAs were washed with binding buffer containing 100 mM (solid line), 150 mM (dashed line), or 200 mM (dotted line) NaCl without any protein to monitor protein dissociation (blue trajectories). The purple dashed line marks the baseline in fluorescence intensity of the Cy3-labeled DNAs prior to protein binding. Each trajectory was averaged over 15 – 20 DNAs. Integrated intensity was normalized to the maximum value in each trajectory. Time zero was defined as the starting point of protein association. All of the dissociation trajectories (blue) have been corrected for photobleaching.

Supplementary Figure 8

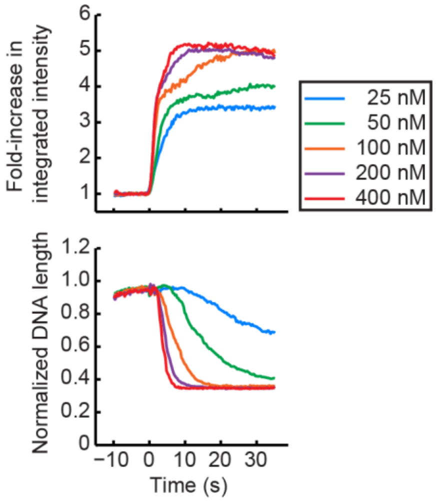

Supplementary Figure 8: Association of wild-type Spo0J to Cy3-labeled DNAs at different protein concentrations. Saturation of the maximum fold-increase in fluorescence intensity occurred at protein concentrations above 100 nM, indicating that the DNAs were coated with proteins. At these same concentrations, DNAs were compacted rapidly all the way to the DNA tether point. These results are consistent with the stabilization of wild-type Spo0J on DNA due to the formation of higher-order bridging interactions. Each trajectory was averaged over 15 – 20 DNAs. Fold-increase in integrated intensity and normalized DNA length were calculated by dividing each trajectory by the values averaged for the first few seconds prior to protein binding. Time zero was defined as the starting point of protein association.

Supplementary Figure 9

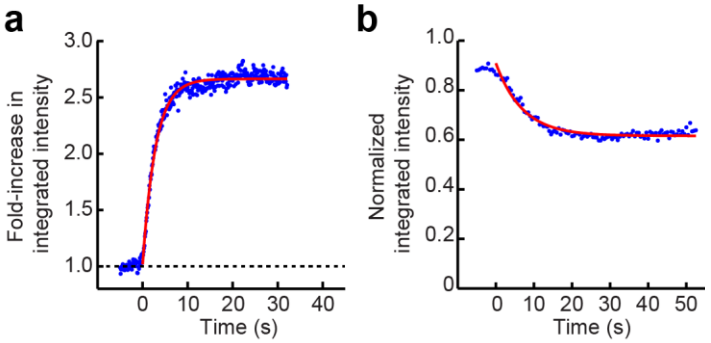

Supplementary Figure 9: Fitting the DNA binding kinetics of Spo0J R82A with the 1:1 Langmuir binding model. (**a**) A representative trajectory showing association of Spo0J R82A (100 nM) in binding buffer containing 150 mM NaCl to Cy3-labeled DNAs. Fold-increase in integrated intensity was calculated by dividing the trajectory by the value averaged for the first few seconds prior to protein binding. Time zero was defined as the starting point of protein association. The fitting parameters shown here (see Methods) are c_0 = 1.02 ± 0.02 (fit ± error estimate), c_1 = 1.65 ± 0.02, and k_{obs} = 0.35 ± 0.01 s^{-1}. (**b**) Trajectory showing dissociation of Spo0J R82A corresponding to (**a**) when washing the DNAs with the same binding buffer but without protein. Integrated intensity was normalized by the maximum value. Time zero was defined as the starting point of protein dissociation. The fitting parameters shown here (see Methods) are A = 0.616 ± 0.002, B = 0.29 ± 0.01, and k_{off} = 0.14 ± 0.01 s^{-1}. Both trajectories shown in (**a**) and (**b**) were averaged over 20 – 30 DNAs.

Supplementary Figure 10

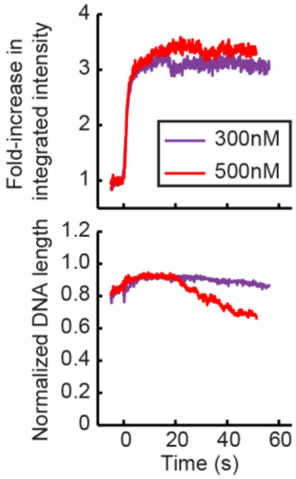

Supplementary Figure 10: Concentrations of Spo0J R82A above 300 nM in binding buffer containing 150 mM NaCl weakly compacted DNA. Each trajectory was averaged over 15 – 20 DNAs. The fold-increase in integrated intensity and normalized DNA length were calculated by dividing each trajectory by the values averaged for the first few seconds prior to protein binding. Time zero was defined as the starting point of protein association.

Supplementary Figure 11

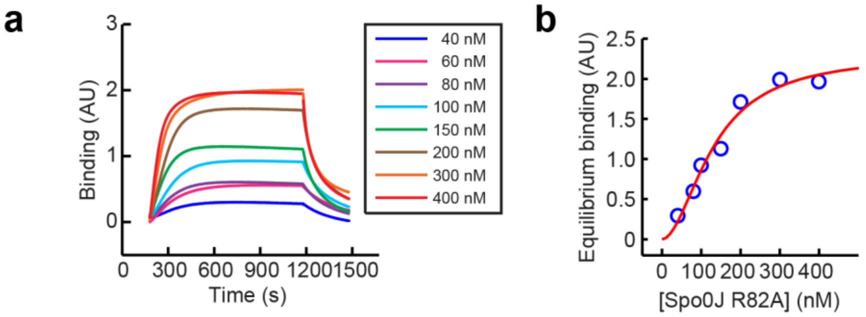

Supplementary Figure 11: Measuring DNA binding affinity (K_d) of Spo0J R82A in binding buffer containing 150 mM NaCl using white light interferometry. (**a**) Trajectories (baseline subtracted) showing association and dissociation of Spo0J R82A with a 39 bp dsDNA at indicated concentrations (see Methods). (**b**) Fitting of the binding response at equilibrium with a Hill equation (see Methods).

Supplementary Figure 12

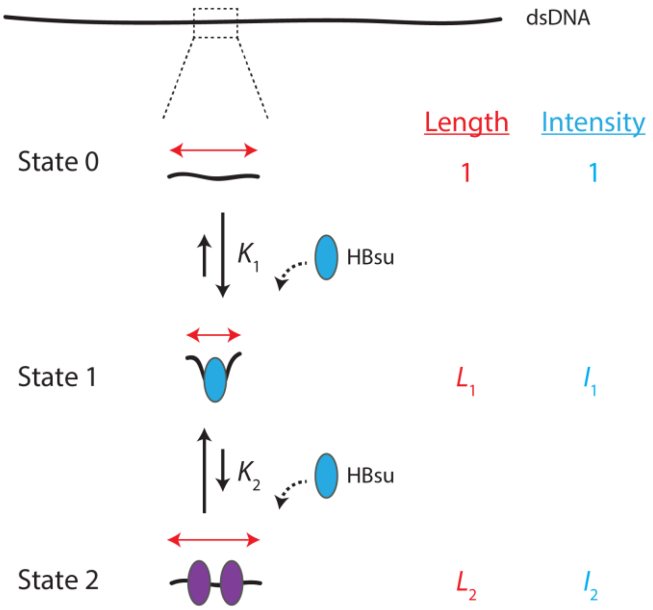

Supplementary Figure 12: Two-state binding model for HBsu. A DNA molecule was modeled as an infinite string of segments, each of which could bind zero, one, or two HBsu dimers. In State 1, HBsu binds to each DNA segment with affinity K_1 and compacts DNA by local bending. In State 2, an additional HBsu dimer can bind to DNA with affinity K_2. The associated proteins might adopt a different conformation on the DNA (purple) to form a nucleoprotein filament, which results in DNA re-extension. Our steady-state measurements indicated that State 1 is more stable than State 2, i.e., $K_1 < K_2$ (Fig. 4). The red arrow marks the length of the DNA segment in each state. Fold change in DNA length and integrated fluorescence intensity relative to the unbound state (State 0) in State 1 and 2 are parametrized as L_1, I_1, and L_2, I_2, respectively.

Supplementary Figure 13

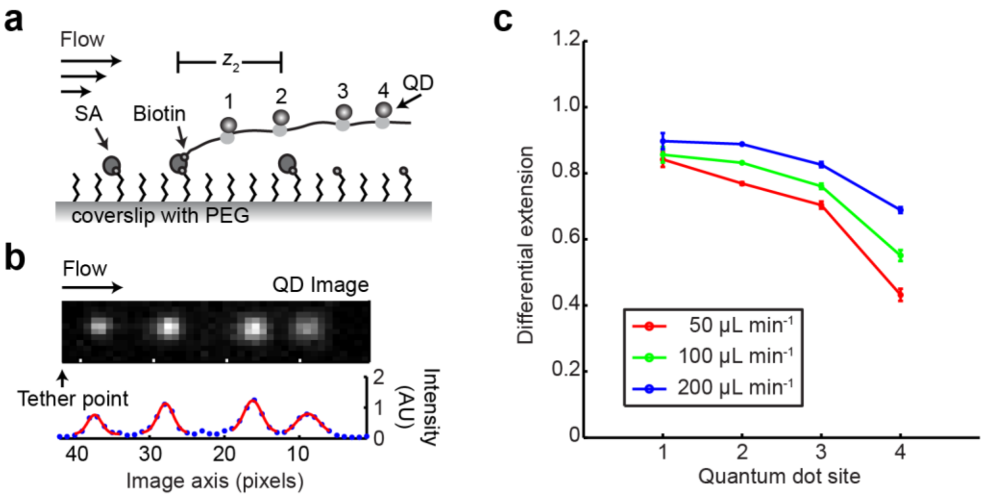

Supplementary Figure 13: Measuring differential extension of a flow-stretched DNA using DNA motion capture assay. (**a**) Schematic (not drawn to scale) showing a 24 kb DNA labeled with four quantum dots (see Methods) and extended under a buffer flow. QD: quantum dot. SA: streptavidin. PEG: polyethylene glycol. z_i is the distance of QD i from the tether point, where i runs from 1 to 4, with i = 1 closest to the tether point (see Methods). (**b**) *Top*: image of a single DNA labeled with four quantum dots extended under a buffer flow at a rate of 200 μL min^{-1}. Tether point of the DNA was determined by SYTOX staining. *Bottom*: one-dimensional projection of the quantum dot image to the axis parallel to the flow direction (blue points) fitted with Gaussian distributions (red lines) to determine the subpixel position of each quantum dot from the tether point. (**c**) Differential extension between the quantum dot sites along the 24 kb DNA while the DNA was extended under different flow rates (see Methods for additional details). Data shown are mean ± s.e.m. measured over 20 – 30 DNAs.

Supplementary Figure 14

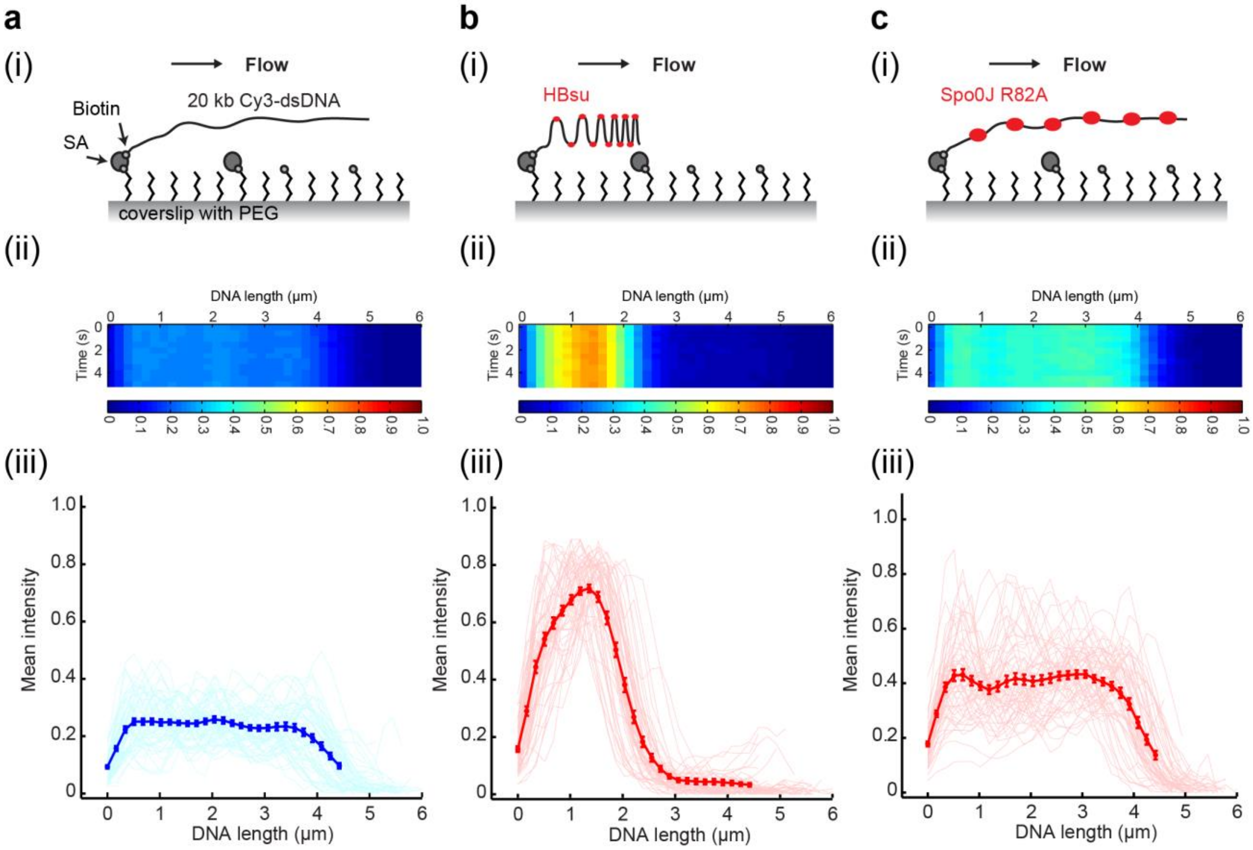

Supplementary Figure 14: Average intensity profile along the length of a Cy3-labeled DNA at steady state. (**a**) Naked DNAs prior to protein binding. (**b**) The same DNAs as shown in (**a**) after being compacted by HBsu (40 nM). (**c**) DNAs coated with Spo0J R82A (50 nM) in buffer containing 150 mM NaCl. (**i**) Schematic of a flow cell (not drawn to scale). SA: streptavidin. PEG: polyethylene glycol. (**ii**) Heat map showing overlaid snapshots of 50 – 60 Cy3-labeled DNAs at steady state. Colors represent normalized fluorescence intensities under the same imaging conditions. (**iii**) Plot of fluorescence intensities along the lengths of DNAs at steady state averaged over a 4 second time window as shown in (**ii**). Profiles of individual DNAs are shown in (**a**) cyan or (**b**) – (**c**) pink and the averaged values with s.e.m. are plotted in (**a**) blue or (**b**) – (**c**) red.

Supplementary Figure 15

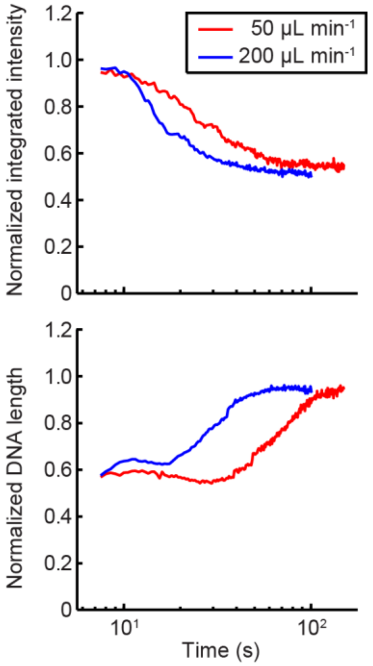

Supplementary Figure 15: Force-dependent biphasic dissociation of HBsu (400 nM). Washing buffer was introduced to the flow cell at indicated flow rates. Each trajectory (log-scale in time) was averaged over 20 – 30 DNAs and was corrected for photobleaching. Integrated intensity (top) and DNA length (bottom) were normalized by the maximum values. Time zero was defined as the starting point of protein dissociation and was shifted by 10 s for visualization.

SUPPLEMENTARY TABLES

Supplementary Table 1: Oligonucleotides used in this study

Number	Name	Sequence
-	BL2	GGGCGGCGACCT/3BioTEG/
oTG043R	parS2_rev (scrambled *parS*)	TTTTTGCTCTCATTGTTAGTCATTTGTCAGATTC AACTG
oTG044F	Bio_parS2_for	/5Biosg/CAGTTGAATCTGACAAATGACTAACAA TGAGAGCAAAAA
oTG489	parS_359_long1F_BioAd	**TGAGGGATATCGAATTCCTGCAGGC**GCTATTC CTCGAGGGAGGTGTC
oTG491	parS_359_long2R_DigAd	**GACGCGAATTATTTTTGATGGCG**GGATCTGCC GCACTGATCTCA
oTG437	5'Bio-TOM-insert	/5Biosg/TGAGGGATATCGAATTCCTGCAGGC
oTG488	Dig_for_oligo1	/5DigN/GACGCGAATTATTTTTGATGGCG

- All oligonucleotides were obtained from Integrated DNA Technologies (IDT) and the sequences are given in the 5' – 3' direction.
- Bold texts in oTG489 and oTG491 indicate adapter sequences that are complementary to oTG437 and oTG488 respectively.

<u>Modifications</u>:

/3BioTEG/ - 3' biotin
/5Biosg/ - 5' biotin
/5DigN/ - 5' digoxigenin

SUPPLEMENTARY REFERENCE

1. Ko, D.S. Photobleaching time distribution of a single tetramethylrhodamine molecule in agarose gel. *J Chem Phys* **120**, 2530-2531 (2004).
